# Public health round-up

**DOI:** 10.2471/BLT.22.011122

**Published:** 2022-11-01

**Sudan virus disease in Uganda**

A team gear up for their shift at the Ebola treatment centre in Mubende district, Uganda where an outbreak of Sudan virus disease was declared on 20 September. As of 12 October, a total of 74 people were reported to have been infected (54 confirmed and 20 probable) across five districts, 39 of whom were reported to have died.

**Figure:**
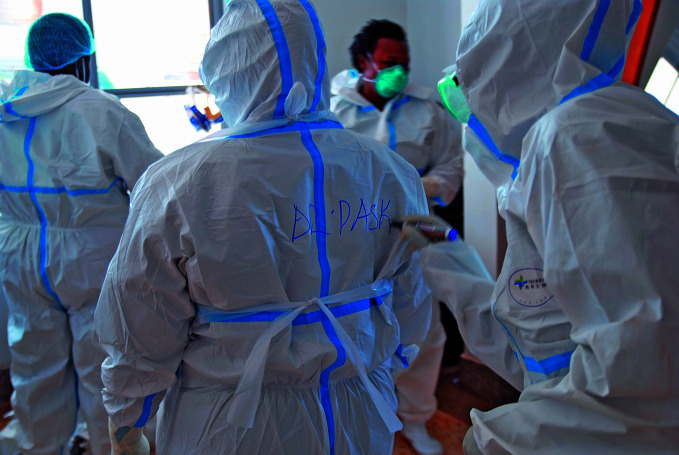
WHO/Vincent D.

## Sudan virus disease in Uganda

On 20 September 2022, Uganda health authorities declared an outbreak of Sudan virus disease caused by Sudan virus, following laboratory confirmation of a patient from a village in the Mubende district in central Uganda.

According to the International Classification of Disease (ICD-11) for filoviruses released in May 2019, Ebola virus disease is now subcategorized depending on the causative virus. Outbreaks of Ebola virus disease caused by Sudan virus are named Sudan virus disease outbreaks. Prior to May 2019 all viruses causing Ebola virus disease were grouped together.

As of 12 October, a total of 74 people were reported to have been infected (54 confirmed and 20 probable) across five districts in central Uganda, 39 of whom were reported to have died. One case had been reported in the capital Kampala which is roughly 150 kilometres east of Mubende.

The risk of further spread through multiple transmission chains was assessed to be high, given that the first deaths occurred in the community. Patients in private facilities and hospitals and other community health services therefore had limited infection prevention and control measures.

The absence of licensed vaccines and therapeutics for prevention and treatment of Sudan virus is also a matter of concern. Six candidate vaccines are currently under development against the Sudan virus, three of which have undergone Phase I or II clinical trials.

The Ugandan government hosted an emergency meeting with neighbouring countries on 12 October to agree on a strategy for collaboration. The World Health Organization (WHO) released US$ 2 million from its Contingency Fund for Emergencies (a pool provided by 16 Member States) to fund response efforts and, in collaboration with partners, is providing additional expertise and resources.

https://bit.ly/3rPy7Or

https://bit.ly/3Eeq993

## Responding to cholera outbreaks

Two Member States declared significant outbreaks of cholera. Between 25 September and 8 October, the Haiti Ministry of Public Health and Population reported 32 laboratory-confirmed cases of *Vibrio cholerae*, and 224 suspected cases from Port-au-Prince and Cité Soleil. As of 12 October, 189 people had been hospitalized, 16 of whom were reported to have died.

The health ministry, WHO and other partners initiated a multisectoral response that included health system support focused on case management and prevention, including vaccination, and efforts to address water, hygiene and sanitation (WASH) challenges. Efforts were also being made to improve security conditions in affected areas under the control of armed gangs.

In Syria, the Ministry of Health declared an outbreak of cholera on 10 September. As of 1 October, 524 confirmed cases had been reported in 11 governorates and a total of 36 people had died. The situation was reported to be evolving rapidly in the affected governorates with cases being reported in new areas.

WHO is working with health partners to contain the outbreak and prevent further spread of infection, shipping 60 tonnes of supplies from its logistics hub in Dubai, enhancing cholera surveillance in high-risk areas, and promoting community awareness.

https://bit.ly/3CTUNne

https://bit.ly/3C9ADnA

## Pakistan flood response

WHO issued an appeal for US$ 81.5 million to fund the Organization’s work in response to Pakistan’s flood-related humanitarian crisis.

As of 12 October, WHO had received just under 20% of the funds requested, contributions coming from Norway, the United Nations Central Emergency Response Fund and WHO’s Contingency Fund for Emergencies.

The funds are being used to treat severely malnourished children, halt outbreaks of malaria, cholera and hepatitis, restart disrupted immunization programmes, repair ruined health centres – which number in the thousands – and carry out other health-related activities.

“Although the waters have stopped rising, the danger is only increasing,” said WHO Director-General Tedros Adhanom Ghebreyesus at a 5 October media briefing. “More than 1500 lives were lost in the floods, but many more could be lost to disease in the coming weeks without a massive and urgent international response.”

https://bit.ly/3VmjQGG

## Dementia R&D blueprint

WHO launched a new Dementia R&D blueprint, the first WHO initiative of its kind relating to a noncommunicable disease. Published on 4 October, the blueprint provides guidance on how to accelerate dementia research and innovation globally with a focus on making research more efficient, equitable and impactful.

Optimizing research and innovation is key to better understanding, preventing and treating the dementia, while at the same time improving the provision of care and support for people with dementia and their carers.

“Although dementia is the 7th leading cause of death globally, dementia research accounts for less than 1.5% of total health research output” said Soumya Swaminathan, WHO’s Chief Scientist.

https://bit.ly/3rWC3wS

https://bit.ly/3CwmJxg

## Boosting genomic surveillance in the Americas

Health authorities of the Region of the Americas agreed on a strategy to boost genomic surveillance of pathogens with pandemic and epidemic potential in the region.

Announced 28 September, the *Strategy on Regional Genomic Surveillance for Epidemic and Pandemic Preparedness and Response* aims to expand and consolidate a regional genomic surveillance network of public health, animal health, and environmental health laboratories.

Efforts will focus on strengthening laboratory capacity to perform genomic sequencing and the reporting systems needed to ensure timely reporting of genomic data. The Pan American Health Organization will provide implementation support to Member States and will also promote the consolidation of a regional genomic surveillance network for epidemic and pandemic preparedness and response.

https://bit.ly/3SRy8Np

## Tackling stress at work

WHO published guidance on mental health at work. Released on 28 September, the guidance recommends actions to tackle risks to mental health that include heavy workloads, negative interpersonal behaviours and other factors that create distress at work.

For the first time WHO recommends training of managers to prevent stress in the work environment and to respond to workers in distress.

A joint WHO/International Labour Organization policy brief was also published on the topic and the two organizations called for concrete actions to address the issue.

https://bit.ly/3SPL3zq

## The mental health of health workers

In related news, a report by the Qatar Foundation, World Innovation Summit for Health, published in collaboration with WHO suggests that at least a quarter of health and care workers are suffering anxiety, depression and burnout symptoms.

Released on 5 October, *Our duty of care: a global call to action to protect the mental health of health and care workers* examines the impact of the coronavirus disease 2019 (COVID-19) pandemic on the mental health of health and care workers mental health and offers 10 policy actions as a framework for immediate follow-up by employers, organizations and policy-makers.

“Well into the third year of the COVID-19 pandemic, this report confirms that the levels of anxiety, stress and depression among health and care workers has become a ‘pandemic within a pandemic,’” said Jim Campbell, WHO Director of Health Workforce.

https://bit.ly/3SYYSfm

**Cover photo**

Jumaa, a 12-year-old boy who lost his right foot a year ago after stepping on a mine, stands before his family’s home in Abu Abdeh village in Aleppo, Syria, on 19 April 2022.

**Figure:**
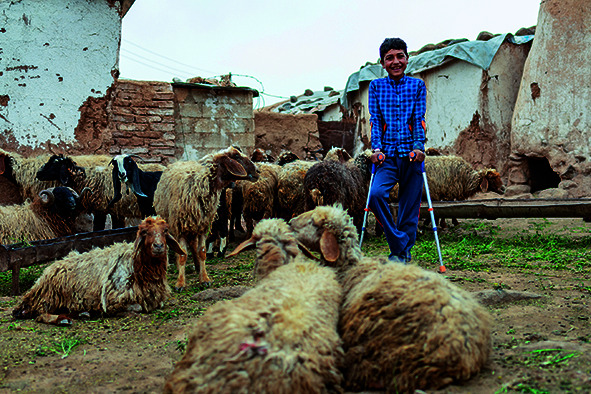
UNICEF/UN0646047/Janji

## Coverage of eye care

WHO launched the first report of progress towards the 2030 targets on effective coverage of eye care. Released on 12 October, the report draws on key results from a comprehensive analysis of population-based eye health surveys and includes the first estimates of effective cataract surgery coverage and effective refractive error coverage at the global level, by WHO region, sex and World Bank income level, and the relative quality gap (the percentage difference between “effective coverage” and “coverage”).

The report serves to highlight key gaps in current data and presents suggestions for additional efforts required to advance surveillance, policies and programmes for increasing the coverage of eye care interventions.

https://bit.ly/3Tghh7g

**Looking ahead**

16–18 November, Sixth session of the Meeting of the Parties to the Protocol on Water and Health. Palais des Nations Geneva Switzerland. https://bit.ly/3CvGCVd

22–24 November, The Healthy Cities Annual Business Meeting and Technical Conference. Copenhagen, Denmark. https://bit.ly/3D1mNG2

24–25 November, Third Global High-Level Ministerial Conference on Antimicrobial Resistance. Muscat, Sultanate of Oman. https://bit.ly/3RITjQV

